# Supplementary material for: Mechanism for Vipp1 spiral formation, ring biogenesis, and membrane repair
Source: Nat Struct Mol Biol. 2024 Nov 11;32(3):571–84. doi: 10.1038/s41594-024-01401-8 (PMC11919738; doi:10.1038/s41594-024-01401-8)
Supplement: Supplementary file 1 — Supplementary Figure 1 [file 41594_2024_1401_MOESM1_ESM.pdf]

nature structural & molecular biology

Supplementary information https://doi.org/10.1038/s41594-024-01401-8

Mechanism for Vipp1 spiral formation, ring biogenesis, and membrane repair

In the format provided by the authors and unedited

A

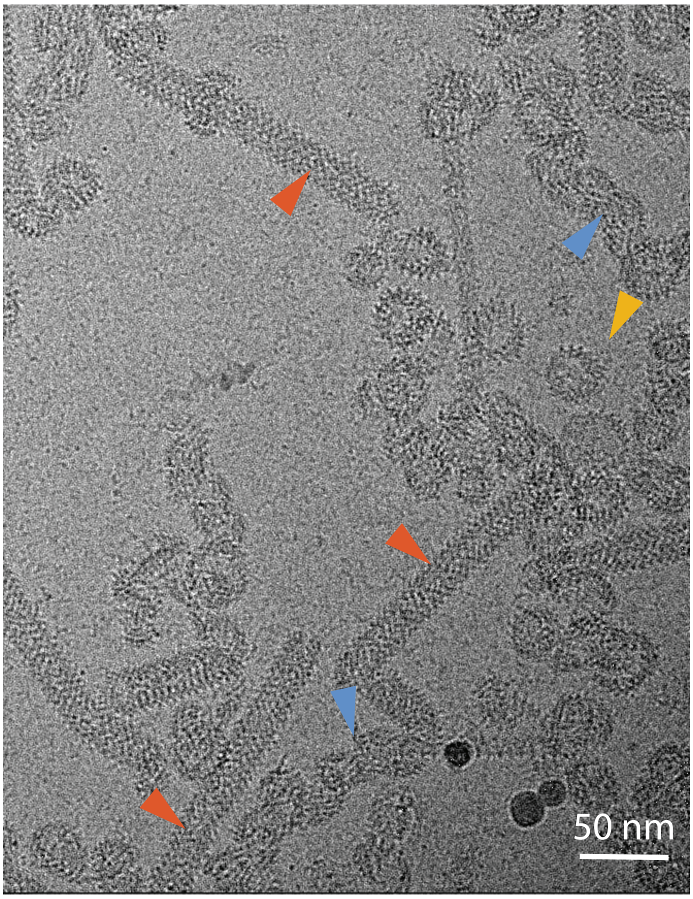

B

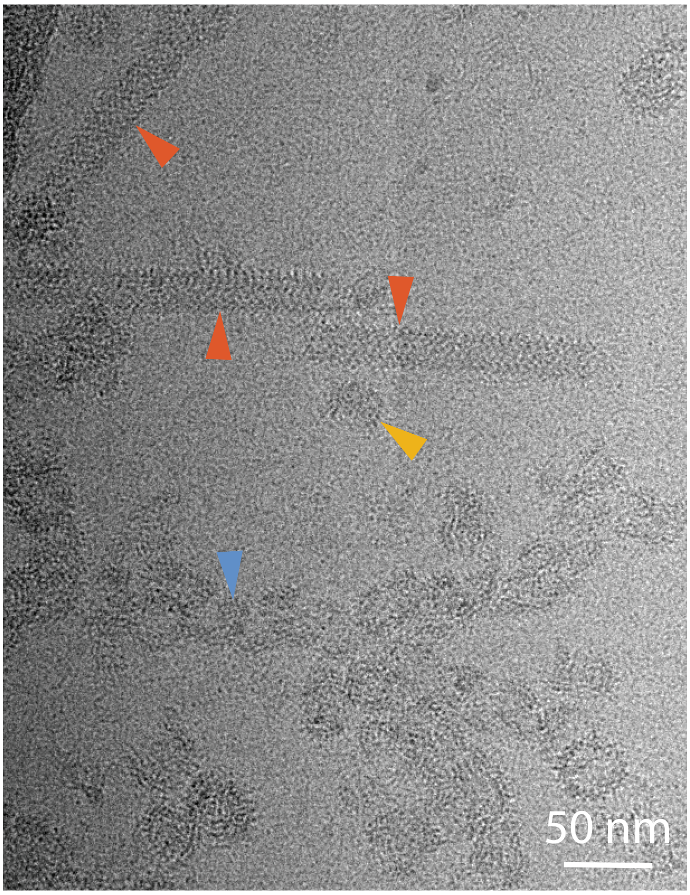

C

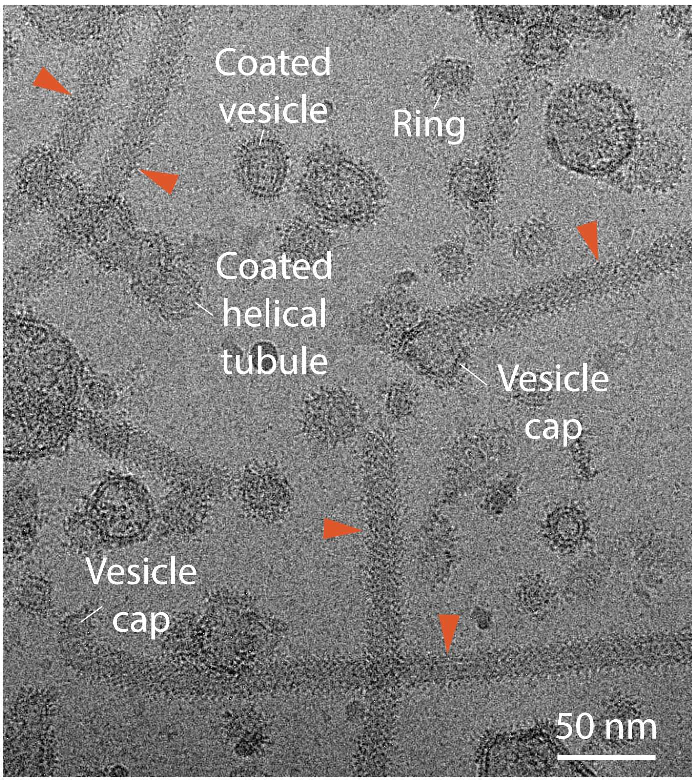

Supplementary Figure 1. Cryo-EM images of Vipp1 polymers.

A, Cryo-EM image showing Vipp1Δα6$_{1-219}$ forming helical filaments, helical-like ribbons, and rings (red, blue and yellow arrows, respectively). Related to Figure 5A.

B, Cryo-EM image showing Vipp1$_{L1}$ forming helical filaments, helical-like ribbons, and rings (red, blue and yellow arrows, respectively). Related to Extended Data Fig. 6A.

C, Cryo-EM image showing Vipp1$_{F197K/L200K_L1}$ forming helical filaments (red arrows) and coated membrane tubules. Related to Figure 6A.
