# Supplementary figures and images for: Mechanism for Vipp1 spiral formation, ring biogenesis, and membrane repair
Source: Nat Struct Mol Biol. 2024 Nov 11;32(3):571–84. doi: 10.1038/s41594-024-01401-8 (PMC11919738; doi:10.1038/s41594-024-01401-8)

Figure 2F

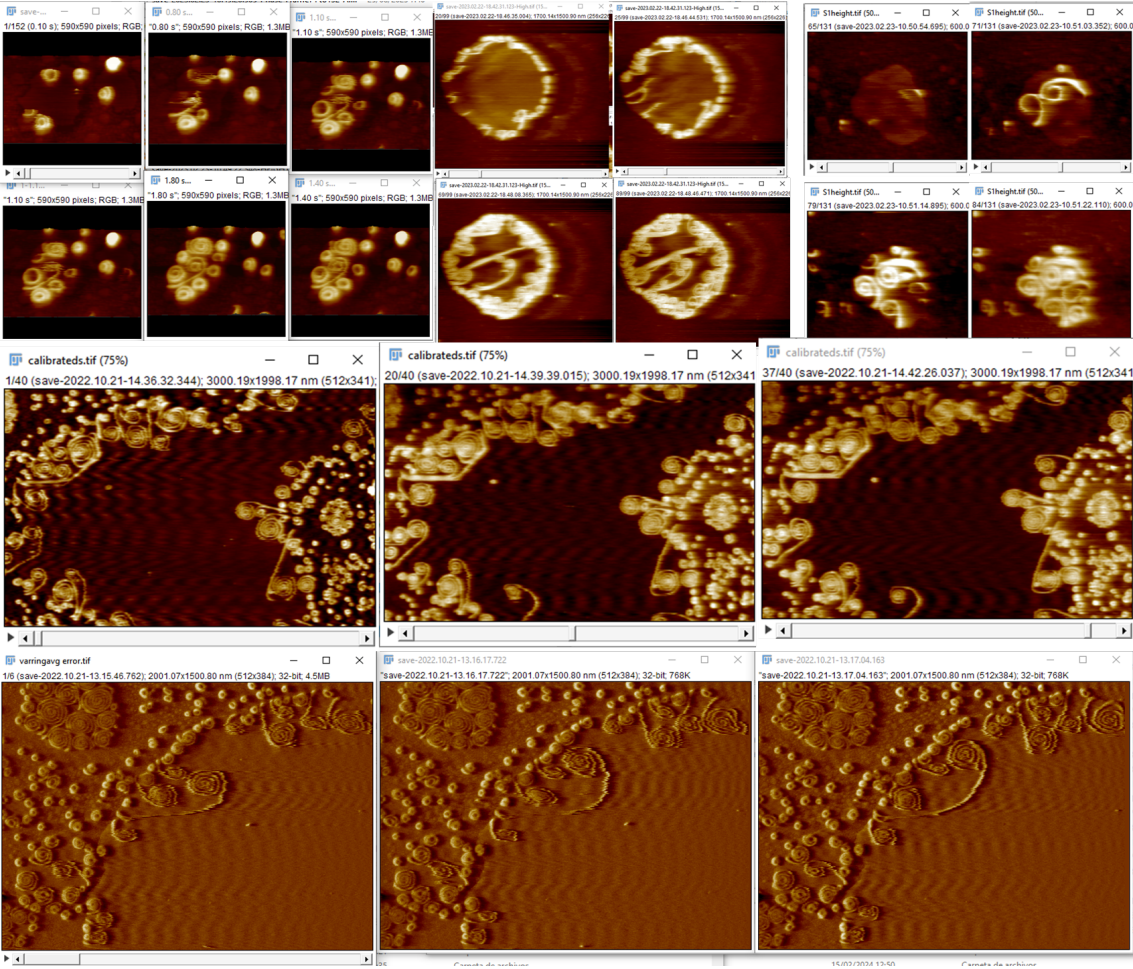

Figure 2G

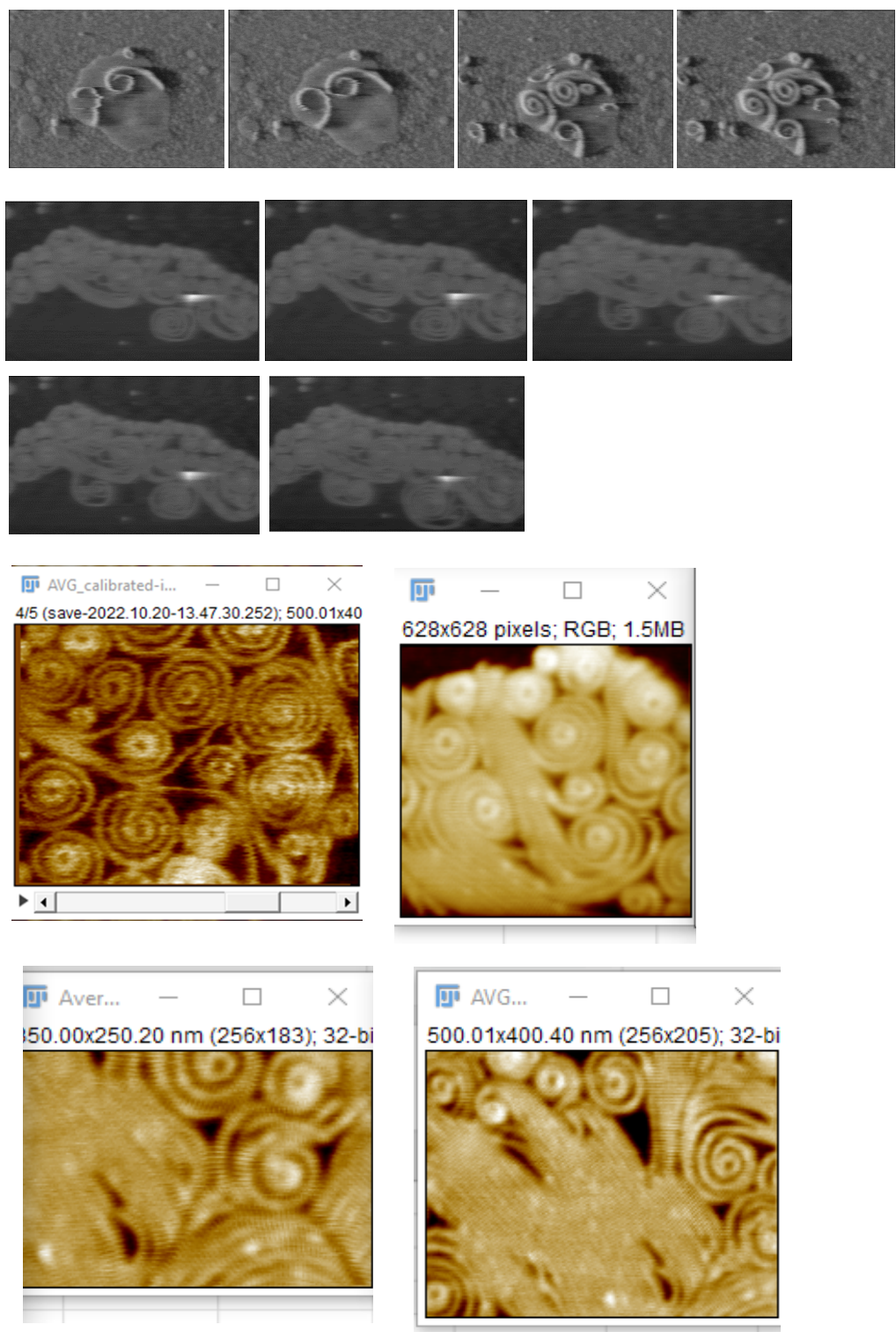

Figure 2H and 2I

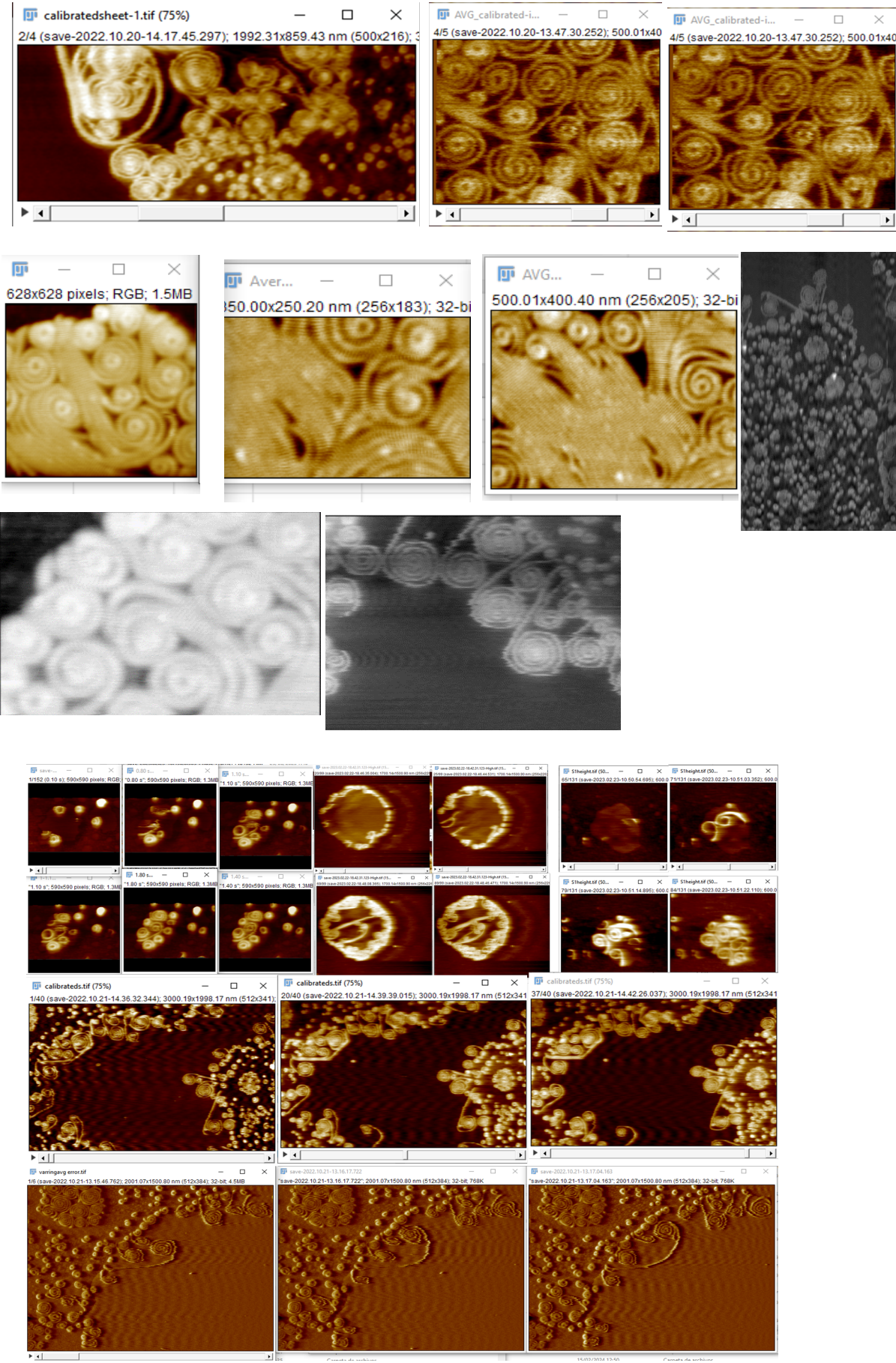

Supplement: Supplementary file 9 — Source AFM images relating to Source Data Fig. 2. [file 41594_2024_1401_MOESM9_ESM.pdf]

Figure 3C

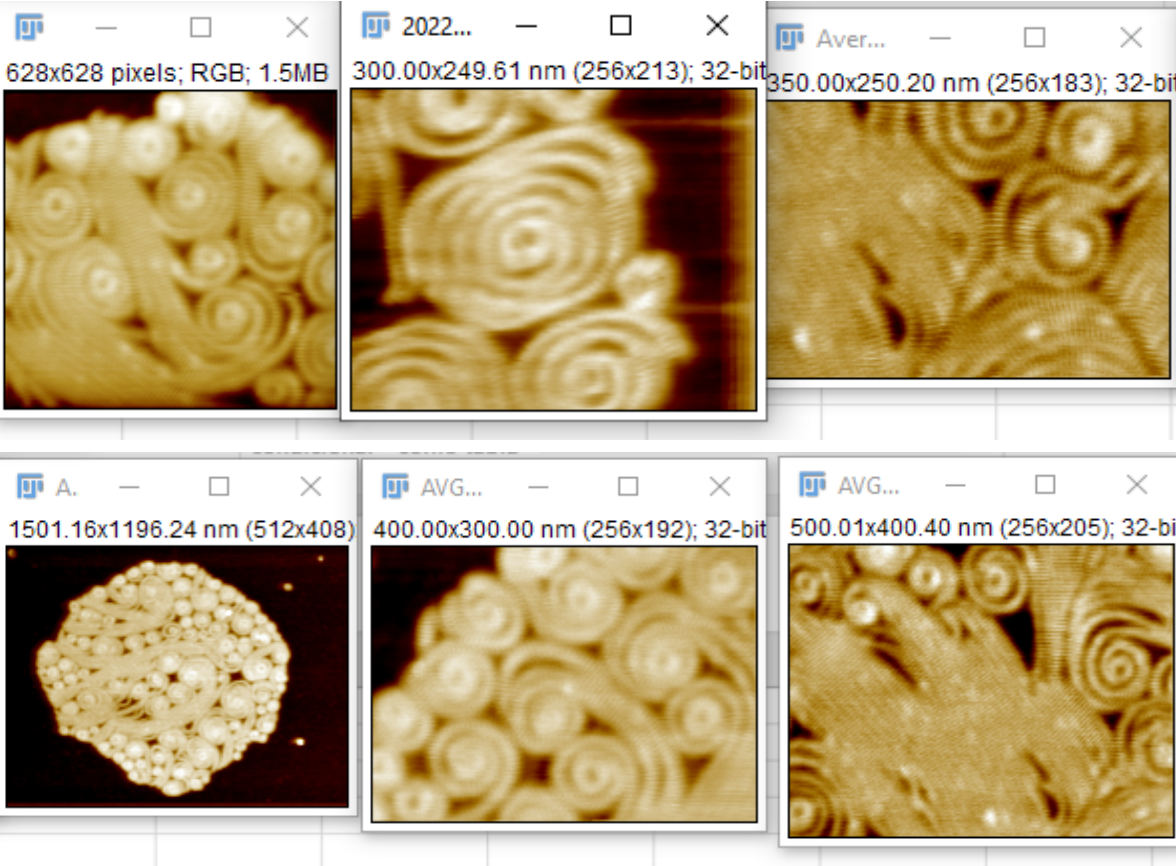

Figure 3F

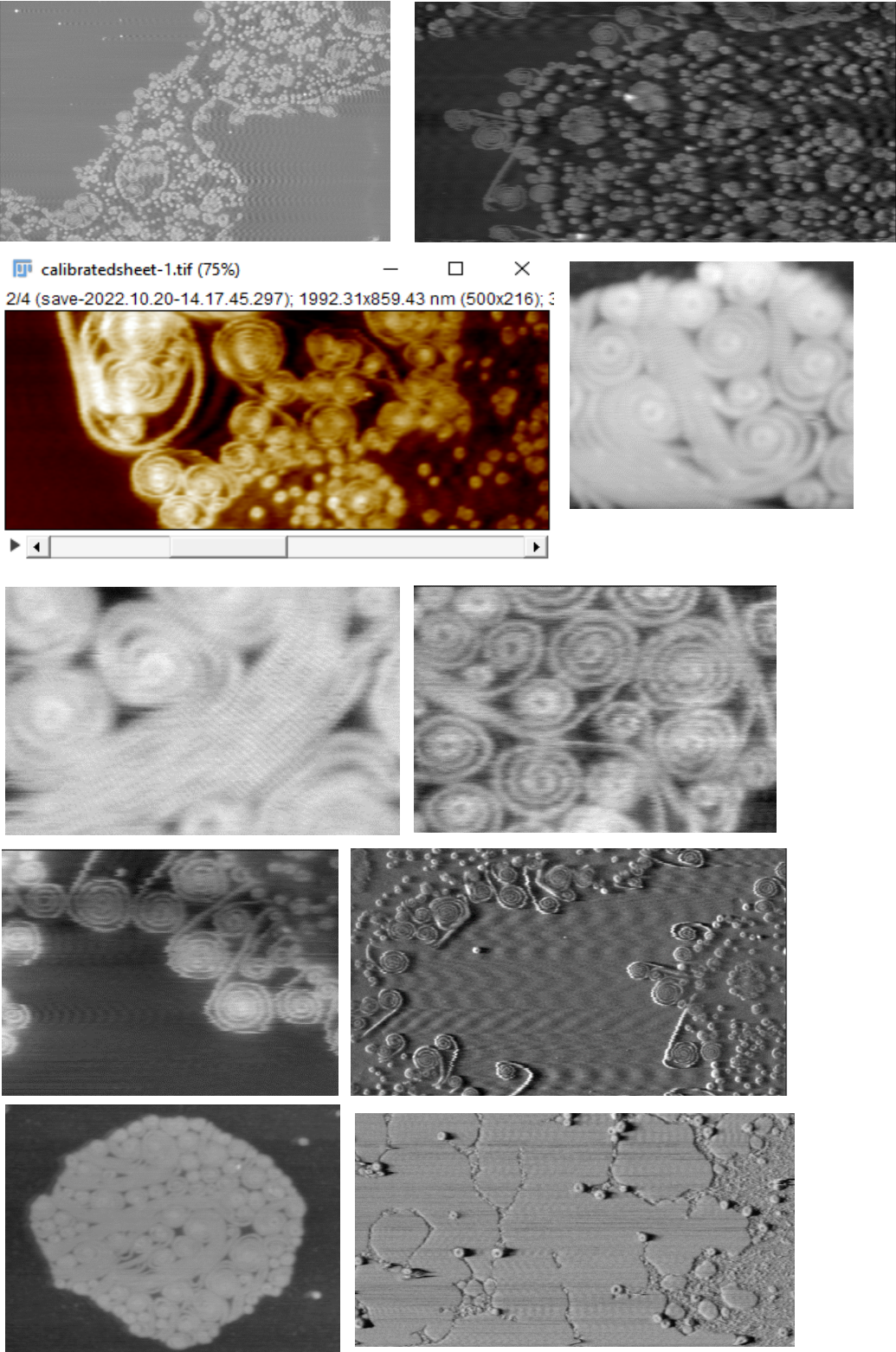

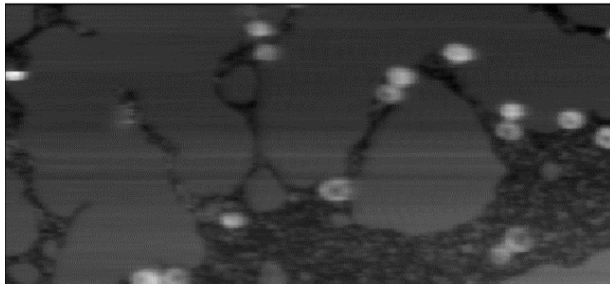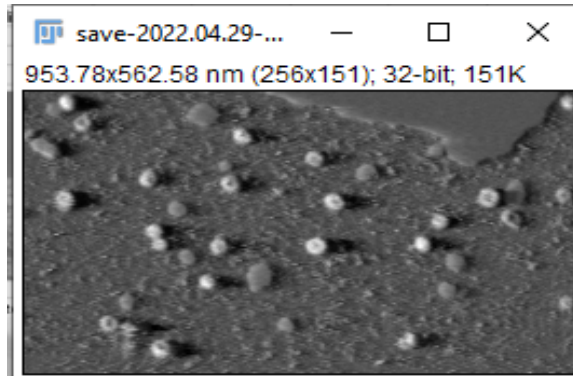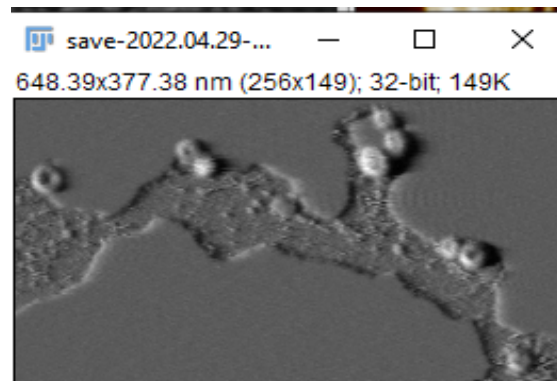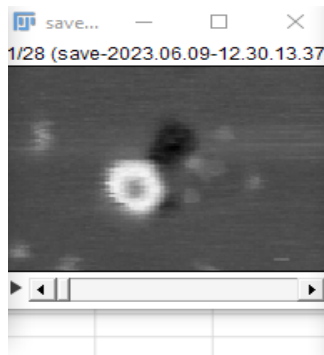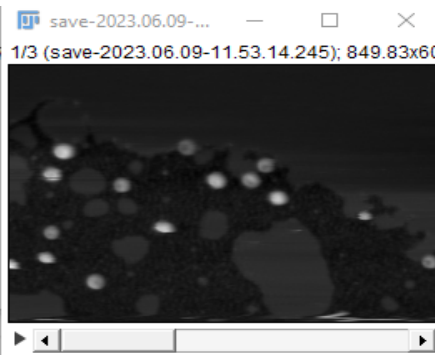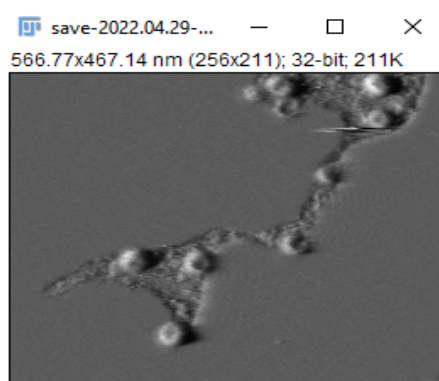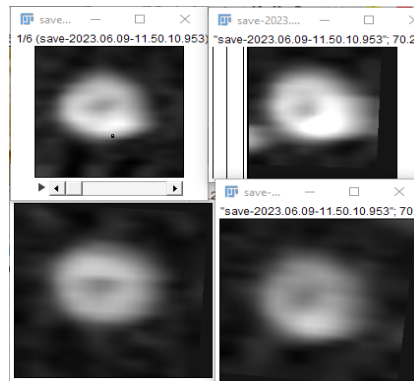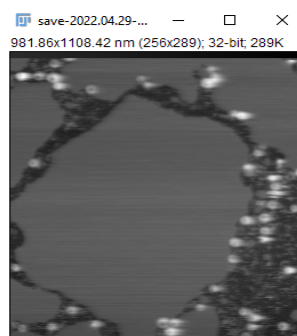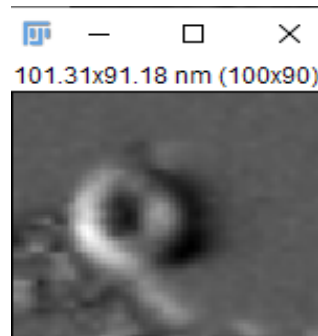

Supplement: Supplementary file 10 — Source AFM images relating to Source Data Fig. 3. [file 41594_2024_1401_MOESM10_ESM.pdf]

Supplementary Figure 1E

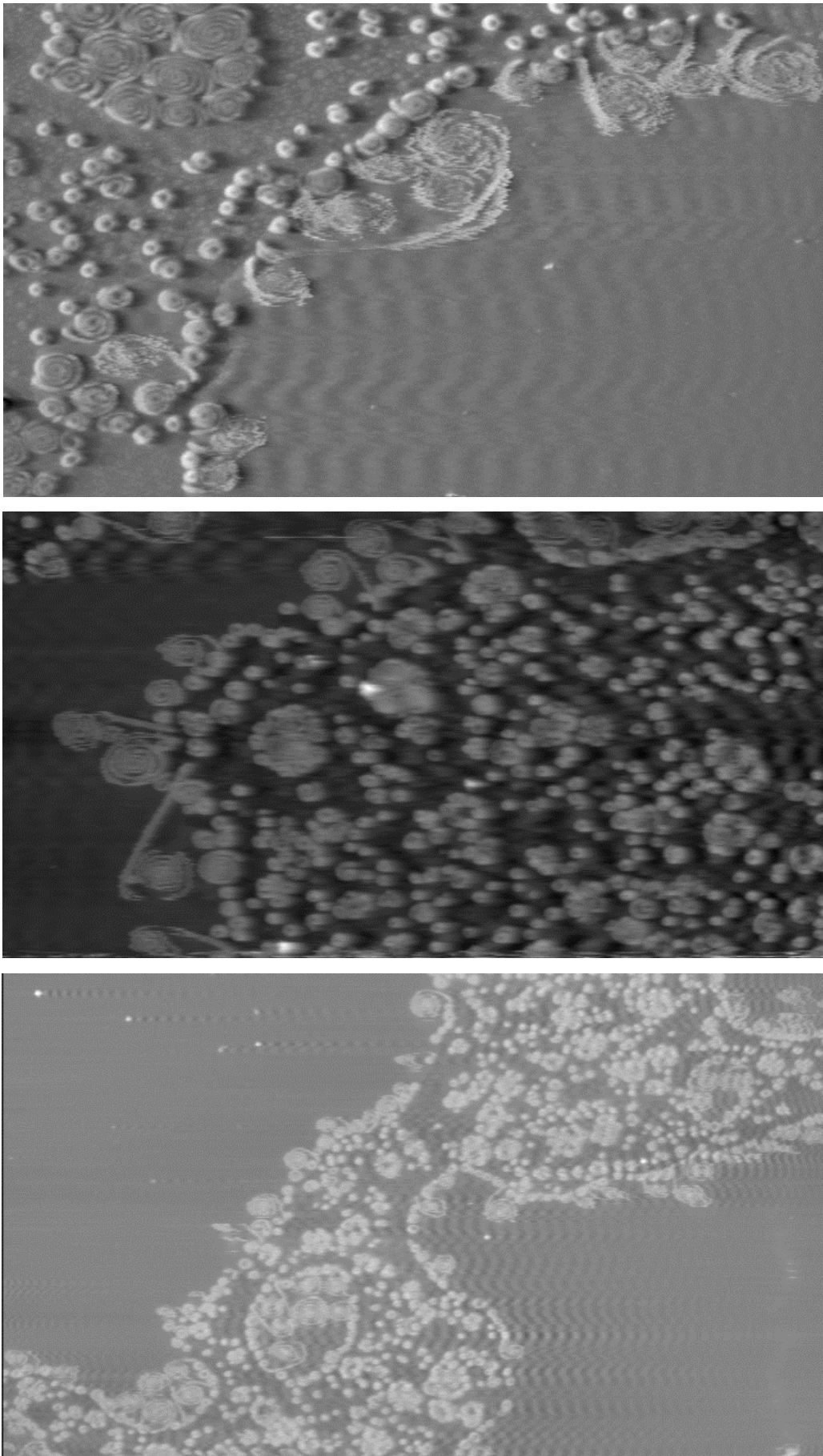

Supplement: Supplementary file 11 — Source AFM images relating to Source Data Extended Data Fig. 1. [file 41594_2024_1401_MOESM11_ESM.pdf]

Supplementary Figure 2C

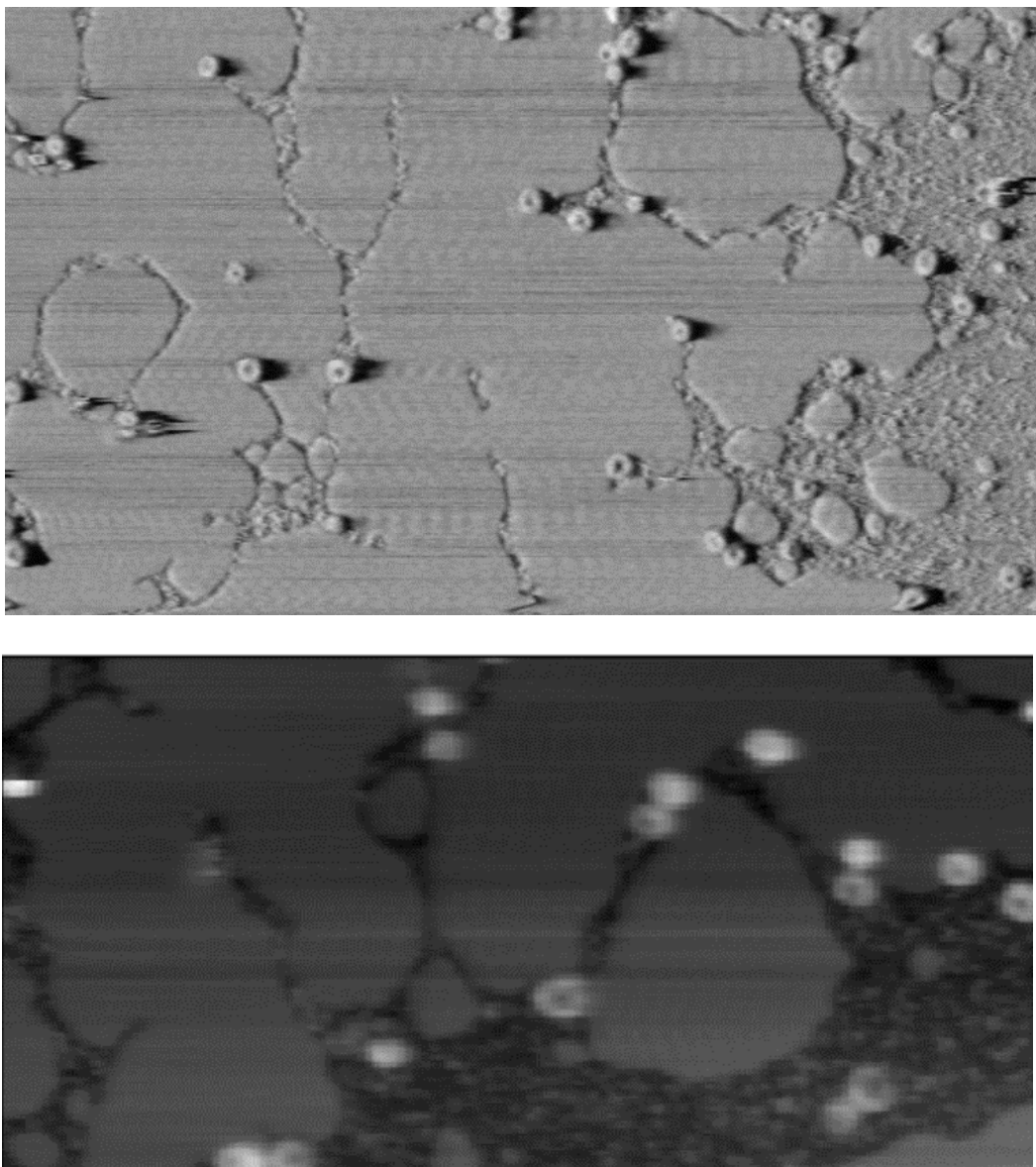

Supplement: Supplementary file 12 — Source AFM images relating to Source Data Extended Data Fig. 2. [file 41594_2024_1401_MOESM12_ESM.pdf]
